# Neutralization and Salt Effect on the Structure and Mechanical Properties of Polyacrylic Acid Gels under Equivolume Conditions

**DOI:** 10.3390/gels7020069

**Published:** 2021-06-09

**Authors:** Yui Tsuji, Mitsuhiro Shibayama, Xiang Li

**Affiliations:** 1 Institute for Solid State Physics, The University of Tokyo, 5-1-5 Kashiwanoha, Kashiwa, Chiba 277-8581, Japan; y.tsuji@issp.u-tokyo.ac.jp; 2 Comprehensive Research Organization for Science and Society, 162-1 Shirakata, Tokai, Ibaraki 319-1106, Japan

**Keywords:** polyelectrolyte gels, osmotic bulk modulus, elasticity, salt, neutralization

## Abstract

The effects of neutralization and salt on the structure and mechanical properties of polyacrylic acid (PAA) gels under equivolume conditions were investigated by small-angle X-ray scattering (SAXS) measurements and tensile tests. We attained the equivolume condition by immersing a piece of PAA gel sample in an ion reservoir containing linear PAA, NaOH, and NaCl at prescribed concentrations (post-ion-tuning). The volume fraction of the linear polymer was set to be the same as that of the gel so as to satisfy the iso-osmotic pressure at the reference state. Various types of reservoirs were prepared by adding NaOH and/or NaCl with different concentrations to the reference reservoir, followed by immersing a PAA gel piece. In the SAXS measurements, a scattering peak appeared, and the scattering intensity at *q* = 0 decreased by neutralization, while the addition of salt increased the scattering intensity. On the other hand, Young’s modulus measured with the tensile test decreased with neutralization; however, it scarcely changed with the addition of salt. The newly developed equivolume post-ion-tuning technique may serve as a new standard scheme to study polyelectrolyte gels.

## 1. Introduction

Polyelectrolyte gels are widely used as water-absorbing materials, cosmetics, and extracellular matrices; they are also a major component constituting our bodies [1]. Recently, the application of polyelectrolyte gels has also expanded to actuators responding to external stimuli, such as pH and temperature changes [2,3], drug delivery vehicles for controlled release [4,5], and structural materials for soft robotics [6,7,8]. The polyelectrolyte gels bear dissociative ion groups in their crosslinked gel networks. The fixed dissociative ion groups on the gel networks exhibit strong Donnan potential [9,10], which is the origin of the high-water absorbing capability of the polyelectrolyte gels.

Many factors affect the physical properties of polyelectrolyte gels, including polymer concentrations, crosslinker densities, spatial and topological heterogeneities, neutralization degrees, salt concentrations, and valence of the salt ions. While the former three parameters are structural factors as crosslinked materials, which strongly depend on how the gels are synthesized, the last three parameters, i.e., neutralization degrees, salt concentrations, and valence of the salt ions, are environmental factors as polyelectrolytes, which can be post-tuned after the gel network is synthesized. These factors also interact intricately with each other. For example, the synthesized network structure of the polyelectrolyte gels strongly depends on the neutralization degrees and salt concentrations in the pregel solution [11]; a pregel solution with the same monomer and crosslinker concentrations may form a gel at one neutralization and salt condition but may not at the other conditions. In addition, when post-tuning the neutralization and salt conditions of the polyelectrolyte gels, the crosslinker densities and polymer concentration may change vastly from their as-prepared state because the gels must be immersed in an aqueous ion reservoir for the post-ion-tuning, and the gels would swell or deswell until they reach a new equilibrium at the condition.

Although tremendous studies have been conducted to elucidate the structures or the physical properties of polyelectrolyte gel in various salt and neutralization conditions [12,13,14,15,16,17,18,19,20,21,22,23,24,25,26,27,28], it is still tricky to independently evaluate the effect of each parameter on the structures and physical properties of the polyelectrolyte gels because of the problems mentioned above. Skouri et al. proposed a scheme to prepare a series of gels with different salt and neutralization conditions by immersing the polyacrylic acid (PAA) gels in a small volume of highly concentrated NaOH and NaCl solutions [17]. Their scheme could minimize the gel volume change by the post-ion-tuning within 20 vol% compared to the as-prepared gels’ volume. However, the actual equivolume condition, i.e., no volume change from the as-prepared state, has not been achieved yet. 

In this study, we achieved the equivolume conditions by immersing the PAA gels into various noncrosslinked PAA solutions with the same polymer volume fraction as the gels but with different salt concentrations and neutralization degrees. We confirmed that the gel volume did not change after the equilibrium condition by evaluating the gel size from microscopy images. SAXS measurements and tensile tests were performed to characterize the nanostructure and the macroscopic elastic properties of these equivolume PAA gels at various neutralization and salt conditions.

## 2. Theory

### 2.1. Osmotic Pressure of Gels

Osmotic pressure is an essential thermodynamic parameter of polyelectrolyte gels, which can be separated into three contributions [29]: (i) the osmotic pressure due to the mixing of the polyelectrolytes and solvent molecules(Π_mix_), (ii) the osmotic pressure due to the mobile ion distributions arising from the Donnan equilibrium (Π_ion_), and (iii) the osmotic pressure due to the elasticity of the crosslinked gel network (Π_el_).

(1) $$\Pi =\Pi_{\mathrm{mix}}+\Pi_{\mathrm{ion}}+\Pi_{\mathrm{el}}$$

### 2.2. Osmotic Pressure Due to Mixing

The Flory–Huggins theory describes the osmotic pressure of mixing for polymer solutions [29]. By regarding a gel as a giant polymer chain, the osmotic pressure of mixing for the gels is given by the Flory–Huggins theory as

(2) $$\Pi_{\mathrm{mix}}=\frac{RT}{v_{0}}\left(-\ln\left(1-\phi \right)-\phi -\chi \phi^{2}\right)$$

where *R* is the gas constant, *T* is the absolute temperature, *v*_0_ is the molar volume of the solvent molecules, *ϕ* is the polymer volume fraction, and *χ* is the Flory–Huggins interaction parameter. 

### 2.3. Osmotic Pressure Due to Imbalanced Mobile Ion Concentrations

The polyelectrolyte gels possess fixed ions on the gel network. When a polyelectrolyte gel is immersed in a solution with mobile ions, mobile ions diffuse into the gel network to balance the chemical potential between the gel and solution. Due to the fixed ions on the gel network, the mobile ion concentrations inside and outside the gel are not equal at the equilibrium, known as the Donnan equilibrium [29]. When the volume of the external solution is much larger than that of the gel, the imbalanced mobile ion concentrations result in the osmotic pressure, Π_ion_, given by

(3) $$\Pi_{\mathrm{ion}}=RT\left(\sqrt{\alpha^{2}z_{p}^{2}{\left(\frac{\phi }{v_{1}}\right)}^{2}+4c_{s}^{2}}-2c_{s}\right)$$

where *α* is the effective charge ratio after considering the counterion condensation, *z*_p_ is the number of chargeable groups per monomer (including the valency of the chargeable groups), *v*_1_ is the molar volume of the monomers, and *c*_s_ is the molar concentration of the added salts. Here, we assume the salt is monovalent. Note that *ϕ*/*v*_1_ is the molar concentration of the polyelectrolytes.

### 2.4. Osmotic Pressure Due to the Network Elasticity

The osmotic pressure due to the network elasticity is given by the classical theory of rubber elasticity [29]. By assuming the isotropic deformation, the osmotic pressure of elasticity is given by

(4) $$\Pi_{\mathrm{el}}=-G_{0}\left(Q^{-1/3}-\frac{Q^{-1}}{2}\right)$$

where *G*_0_ is the shear modulus at the as-prepared state, and *Q* is the swelling ratio compared to the as-prepared gel volume.

## 3. Results and Discussion

**Figure 1:**
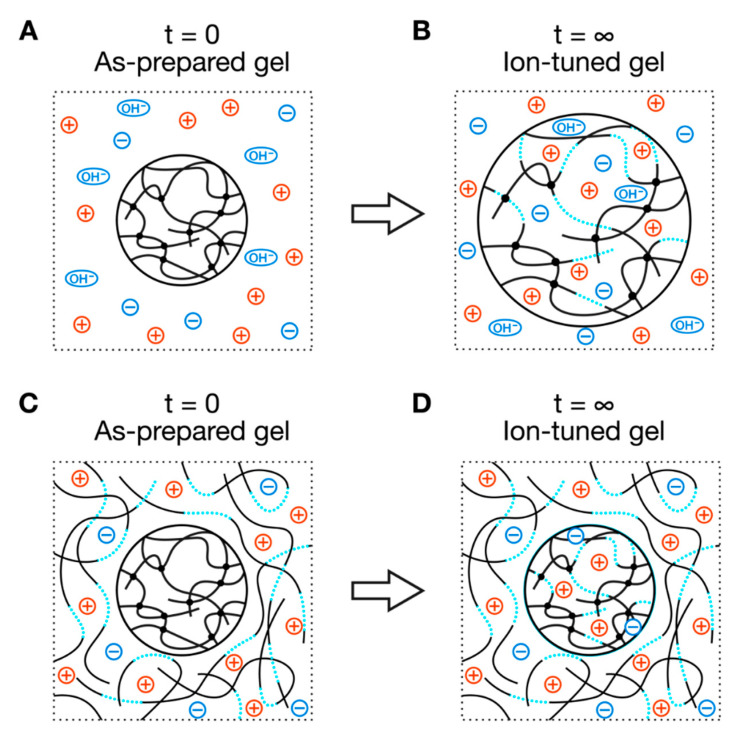
Scheme to prepare the polyelectrolyte gels with the same basic network structures but with different neutralization degrees and salt concentrations. (**A**,**B**) conventional scheme without linear ionizable polymer in the ion reservoir and (**C**,**D**) with linear ionizable polymer in the ion reservoir. The blue dotted curves and black solid curves denote charged polymer segments and noncharged polymer segments, respectively. Circles with red plus symbol and blue minus symbol represent mobile cations and anions, respectively.

To evaluate the effect of the charge density and salt concentration on the nanostructure and mechanical properties of polyelectrolyte gels, we needed to prepare a series of polyelectrolyte gels with the same basic network structures, e.g., crosslinker density, polymer concentration, and spatial and topological heterogeneities, but with different neutralization degrees and salt concentrations. As the synthesis conditions, such as neutralization degrees and salt concentrations, drastically affect the initial networks of the polyelectrolyte gels [11], we prepared all polyelectrolyte gels at the same initial condition. We then post-tuned the gels’ neutralization degrees and salt concentrations by immersing the gels in a series of solutions with different neutralization degrees and salt concentrations (ion reservoir) (Figure 1). In a conventional scheme, the diffusion of ions from the ion reservoir would change the neutralization degrees and salt concentrations in the gels (Figure 1A). As a result, the gels would swell largely at the ion equilibrium (Figure 1B) because the osmotic pressure of mixing in the gel (Equation (2)) is larger than that of the reservoir (ΔΠ_mix_ = Π_mix_(gel) − Π_mix_(res) = Π_mix_(gel) − 0 > 0). The swelling of the gels could change the aforementioned basic gel structures and hinder the goal of our study. To balance these osmotic pressures between the gels and ion reservoirs, we added linear polyelectrolytes with a large molecular weight to the ion reservoir with a polymer concentration the same as the as-prepared polyelectrolyte gels (Figure 1C,D). In such ion reservoirs, the gels’ volume is expected to return to its initial value at equilibrium.

As a model polyelectrolyte gel, we studied the polyacrylic acid (PAA) gels in this study. We synthesized PAA gels by radical copolymerization of acrylic acid (monomer) and N,N′-methylenebisacrylamide (crosslinker) in pure water. The monomer concentration was 15 wt%, and the crosslinker concentration was 0.4 mol% to the monomers. In the as-prepared PAA gels, all monomers were present as acids, i.e., zero neutralization degree (N0), and no additional salts were added (S0). Therefore, the as-prepared gels were in the N0S0 condition. Here, the capital N denotes the neutralization degree, and the capital S denotes the salt concentration. To change the neutralization and salt conditions in the PAA gels, the as-prepared N0S0 gels were immersed in a series of PAA solutions (ion bath) with the same polymer concentration (15 wt%) but different neutralization degrees (N: 0, 50, and 100%) and salt concentrations (S: 0, 250, and 500 mM). We used linear PAA with molecular weight of 250,000 g mol^−1^ to prepare the PAA solutions. The neutralization degrees and salt concentrations in the PAA solutions were tuned by adding sodium hydroxide and sodium chloride. The volume of the PAA solutions immersing the gels were at least 20 times larger than those of the gels; the neutralization degrees and salt concentration remained almost unchanged after the ion equilibrium.

### 3.1. Volume Change during the Post-Ion-Tuning

**Figure 2:**
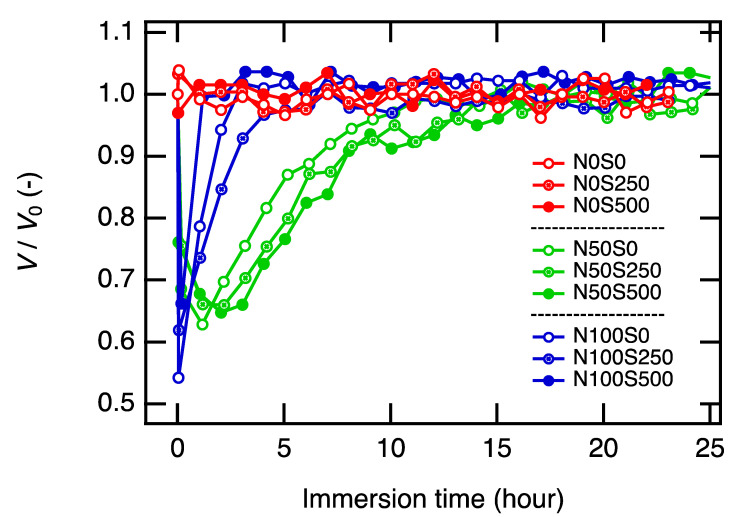
Volume change of the as-prepared N0S0 gels immersed in nine PAA solutions with different neutralization degrees (N: 0, 50, and 100%) and salt concentrations (S: 0, 250, and 500 mM). All PAA solutions have the same polymer concentration as the as-prepared gel (15 wt%). The volume of immersed gels (*V*) was normalized by their as-prepared volume (*V*_0_).

We monitored the volume change of the gels immersed in the different PAA solutions (Figure 2). While the non-neutralized gels (N0 series) remained unchanged throughout the observation, the neutralized gels (N50 and N100 series) shrunk first and then swelled to return to the initial volume. We have confirmed that the neutralization degrees and the salt concentrations in the equilibrated equivolume PAA gels were the same as the corresponding PAA solutions by a series of SAXS experiments, and we will discuss this later. The neutralization degrees and the salt concentrations in the gels were properly post-tuned while the basic network structures, i.e., crosslinker density, polymer concentrations, and spatial and topological heterogeneities, remained the same.

Here, we focus on the discussion of the volume change of the gels. First, let us discuss the gels immersed in the non-neutralized PAA solutions, N0 series. In the case of the N0S0 condition, we did not expect any change in the gel volume because the major osmotic pressures should balance between the PAA gel and the PAA solution (reservoir) from the beginning (ΔΠ_mix_ = Π_mix_(gel) − Π_mix_(res) = 0, ΔΠ_mix_ = Π_ion_(gel) − Π_ion_(res) = 0), and the net osmotic pressure difference between the PAA gels and PAA solutions should be zero, ΔΠ = ΔΠ_mix_ + ΔΠ_ion_ + Π_el_ ≅ 0. As expected, no volume change was observed. Note that the elastic term is negligible in the *as-prepared* gels when crosslinking density is low [30,31], and we did not observe the effect of Π_el_ in this study when the gel volume was close to the as-prepared value.

**Figure 3:**
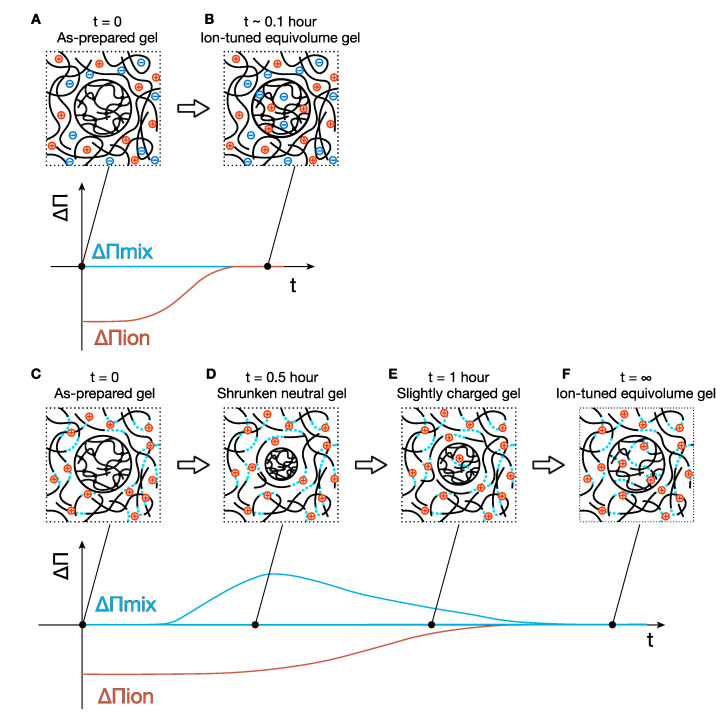
Illustration of the post-ion-tuning process. (**A**) An N0S0 gel is immersed in an N0S500 solution, and (**B**) in a short time (~0.1 h), the gel reaches equilibrium before the gel could take a volume-change response because of the quick ion diffusion. (**C**) An N0S0 gel is immersed in an N50S0 solution. (**D**) The gel shrinks, and the acrylic acid groups in the gel network do not dissociate yet because of the slow dissociation process of PAA. (**E**) The acrylic acid groups in the gel are slightly charged, and the gel swells a bit. (**F**) Finally, the neutralization degree and the salt concentration in the gel become the same as the reservoir, and the gel returns to its initial volume. The graph below each conceptual image of gels indicates the difference of the mixing and ionic osmotic pressure between the gel and reservoir qualitatively.

Concerning N0S250 and N0S500 conditions, we expect the gel to shrink in the beginning because ion concentrations in the PAA solutions were higher than those in the gels, which results in an imbalanced osmotic pressure, i.e., ΔΠ_ion_ = Π_ion_(gel) − Π_ion_(res) < 0, (Figure 3A). The negative net osmotic pressure difference (ΔΠ = ΔΠ_mix_ + ΔΠ_ion_ + Π_el_ ≅ ΔΠ_ion_ < 0) results in a water flow from the gel to the reservoir to minimize the difference in the ion concentrations. However, we did not observe such shrinking of the gels in the N0S250 and N0S500 conditions. The faster diffusion of the ions (*D*~1 × 10^−9^ m^2^ s^−1^) [32] than that of the polymers (*D*_coop_~1 × 10^−11^ m^2^ s^−1^) [33] is highly likely the cause of the no-volume-change process in these conditions. The ion equilibrium is likely already achieved before the gel can take a macroscopic volume-change response.

Next, we move on to the neutralized conditions, N50 and N100. As the ion distribution reaches equilibrium much faster than the gel can take a volume-change response, the observed gels’ shrinking in PAA solutions N50 and N100 series must be attributed to other reasons. A possible account is the slow neutralization process of the PAA chains in the gels. The slow ion dissociation on the polyelectrolytes has been reported in previous studies [34]. To incorporate the slow ion dissociation kinetics, we have derived the osmotic pressure of ions when a polyelectrolyte gel is immersed in a polyelectrolyte solution (Appendix A). According to Equations (A4) and (A5), we can roughly approximate the difference in Π_ion_ between the PAA gels and the PAA solution reservoir as

(5) $${\Delta \Pi }_{\mathrm{ion}}\approx RT\left(\alpha_{\mathrm{gel}}\left(t\right)-\alpha_{\mathrm{res}}\right)z_{p}\frac{\phi }{v_{1}}$$

where *α*_gel_(*t*) and *α*_res_ are the effective charge fraction of the PAA gels at time *t* since the gel is immersed in the reservoir. *α*_res_ does not depend on time because the volume of the reservoir is much larger than that of the gels, and the decrease of ions in the reservoir is negligible. In the case that the PAA strands in the gels were not yet neutralized (*α*_gel_(*t*) < *α*_res_), ΔΠ_ion_ takes a negative value, and the net osmotic pressure difference also takes a negative value (ΔΠ = ΔΠ_mix_ + ΔΠ_ion_ + Π_el_ ≅ ΔΠ_ion_ < 0), resulting in water flow from the gel to the external solution and shrinking of the gels (Figure 3C,D). 

The shrinking of the gels leads to an increase of the mixing osmotic pressure (ΔΠ_mix_ > 0) and elastic osmotic pressure (Π_el_). At some point, the increases in ΔΠ_mix_ and Π_el_ compensate for the decrease in ΔΠ_ion_ so that the net osmotic pressure difference becomes zero, ΔΠ = ΔΠ_mix_ + Π_el_ + ΔΠ_ion_ = 0; the gels stop shrinking (Figure 3D). For neutral gels, this will be the equilibrium state, and no more volume change will happen. However, for polyelectrolyte gels with a slow dissociation process, the polyelectrolyte chains of the gels gradually release protons and become more charged, leading to a recovery in ΔΠ_ion_(*t* = *t*) > ΔΠ_ion_(*t* = 0). The balance between ΔΠ_mix_ and ΔΠ_ion_ breaks, and the gels will start to swell (ΔΠ = ΔΠ_mix_ + Π_el_ − ΔΠ_ion_ > 0) (Figure 3E). When the PAA gel is charged as much as the PAA chains in the reservoir, e.g., ΔΠ_ion_ = 0, the gel recovers to the initial volume to minimize the osmotic pressure of mixing, e.g., ΔΠ_mix_ = 0 (Figure 3F). The reswelling rates of the gels in the PAA solutions N50 series were much slower than those in the N100 series, suggesting that the dissociation rate in the neutral pH conditions (N50) is considerably slower than in basic pH conditions (N100). Such a difference could be attributed to weak acid characteristics, but we could not find a study to discuss the dissociation kinetics at different pH conditions. A more detailed discussion for the shrinking and swelling behavior of the gels is out of the scope of this study, and we finish the discussion for this topic here.

### 3.2. Structural Analysis of the Post-Ion-Tuned Equivolume Gels

**Figure 4:**
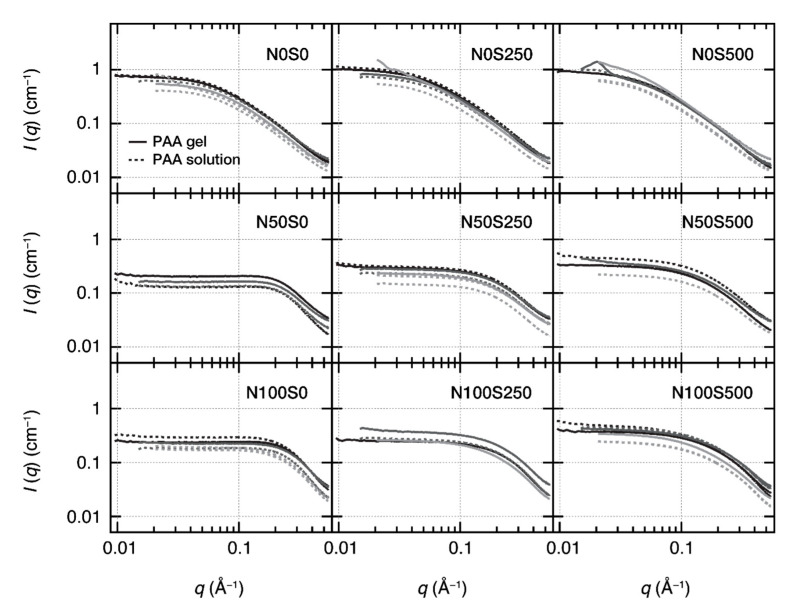
SAXS profiles of the equilibrated equivolume PAA gels (solid curves) and the corresponding noncrosslinked PAA solutions (dotted curves) with different neutralization degrees and salt concentrations. Each panel shows three independent SAXS experiments, where gels and polymer solutions were prepared on each occasion. The profiles with obvious problems were removed.

To evaluate the nanostructures of PAA gels at each neutralization and salt condition, we performed small-angle X-ray (SAXS) measurements. Figure 4 shows the SAXS profiles of PAA gels and PAA solutions in each condition. We performed three independent SAXS measurements to confirm the reproducibility of the gel synthesis and the post-ion exchange processes. The profiles with obvious problems were removed. 

As the neutralization degrees and salt concentrations will largely affect the small-angle scattering profiles of the polyelectrolytes [13], the SAXS profiles of the PAA gels will be similar to those of the PAA solutions (reservoir) only when the post-ion-tuning completes. Figure 4 shows the SAXS profiles at each condition. The gel SAXS profiles are consistent with the sol profiles in each panel within the experimental errors, suggesting that the neutralization degree and the salt concentration in each gel are the same as those in each corresponding PAA solution. This result confirms the completion of the post-ion-tuning, as observed in Figure 2. We also measured the newly prepared PAA solutions and the PAA solutions used for the post-ion-tuning, respectively; no difference was observed in their scattering profiles, indicating that the PAA solutions properly worked as the ion reservoir as we expected.

The slight vertical shifts of the profiles in each panel are due to the uncertainty in the sample thickness in our sample environments, which is known to cause ±20% shifts for the solution samples. The vertical shifts for the gel samples (solid lines) were much smaller than the solutions (dotted lines) because the mold determines the gel thickness. However, as the vertical shifts do not affect the shape of the profiles, they do not affect the discussion in this study. 

**Figure 5:**
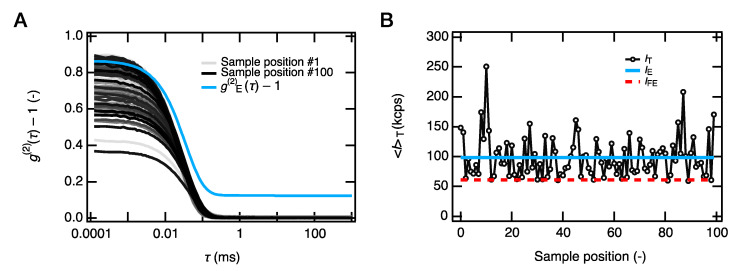
Spatial heterogeneity in a length scale of light scattering. The measurements performed at *q* = 0.00141 Å^−1^. (**A**) DLS intensity correlation functions (*g*^(2)^(*τ*) − 1) at 100 randomly chosen sample positions and the ensemble-averaged correlation function (*g*^(2)^_E_(*τ*) − 1). (**B**) Time-averaged scattering intensity at the 100 sample positions, i.e., stationary laser speckles. The solid blue line indicates the ensemble-averaged scattering intensity (*I*_E_), and the dashed red line shows the time fluctuating component (*I*_FE_) of *I*_E_.

The excellent consistency between the PAA gel and solutions in each condition also suggests a relatively homogeneous network formation in the size range of the SAXS measurements (1.3 Å < 2π/*q* < 62.8 Å). The anomalous fractal small-angle scattering at *q* < 0.05 Å^−1^, representing the heterogeneous networks [35], was not observed in Figure 4. However, the dynamic light scattering experiments and laser speckle tests showed that the as-prepared PAA gels are highly heterogeneous in the light scattering size regime (2π/*q* = 336 nm) (Figure 5a). Typical scattering results for the spatial heterogeneities, such as the position-dependent intensity correlation curves (*g*^2^(*τ*) − 1), a nonrelaxing component in the ensemble-averaged correlation curve (*g*^2^_E_(*τ*) − 1), and the laser speckles, were all observed (Figure 5b). The overlap of the scattering profiles of the heterogeneous gels and polymer solutions in *q* > 0.01 Å^−1^ was reported in the previous SAXS and small-angle neutron scattering (SANS) studies for the gels with low crosslinker densities [36,37]. The consistency of the gel’s SAXS profiles with that of polymer solutions is likely due to the same reason, as the crosslinker density of the PAA gels used here was as low as 0.4 mol% to the monomers.

In a polymer solution or gel, the scattering intensity at *q* → 0, *I*(0), is inversely proportional to the osmotic bulk modulus *K*_os_ = *ϕ*(∂Π/∂*ϕ*) for polymer solutions [38] and to the longitudinal osmotic bulk modulus *M*_os_ = *K*_os_ + 4*G*/3 for gels [33]. Here, *G* is the shear modulus of the gels. The net osmotic pressure of the polyelectrolyte solutions is given as Π = Π_mix_ + Π_ion_, whereas the net osmotic pressure of the polyelectrolyte gels has an additional term Π_el_ (Equation (1)). However, because the contribution of the elastic term in the gel is negligible when the gels are in the as-prepared volume (see our discussion for Figure 2), we can approximate the net osmotic pressure of the equivolume gels as Π ≅ Π_mix_ + Π_ion_, which is the same with the expression for the polyelectrolyte solutions. As long as the gel volume is close to the as-prepared state, we do not need to distinguish the gel and solution samples in the discussion for *I*(0). The osmotic bulk modulus of the mixing (*K*_mix_) and ions (*K*_ion_) are given as

(6) $$K_{mix}=\frac{RT}{v_{0}}\left(\frac{1}{1-\phi }-1-2\chi \phi \right)\phi$$

(7) $$K_{ion}=RT\frac{\alpha^{2}z_{p}^{2}\;{\left(\frac{\phi }{v_{1}^{2}}\right)}^{2}}{\sqrt{\alpha^{2}z_{p}^{2}\;{\left(\frac{\phi }{v_{1}^{2}}\right)}^{2}+4c_{s}^{2}}}$$

**Figure 6:**
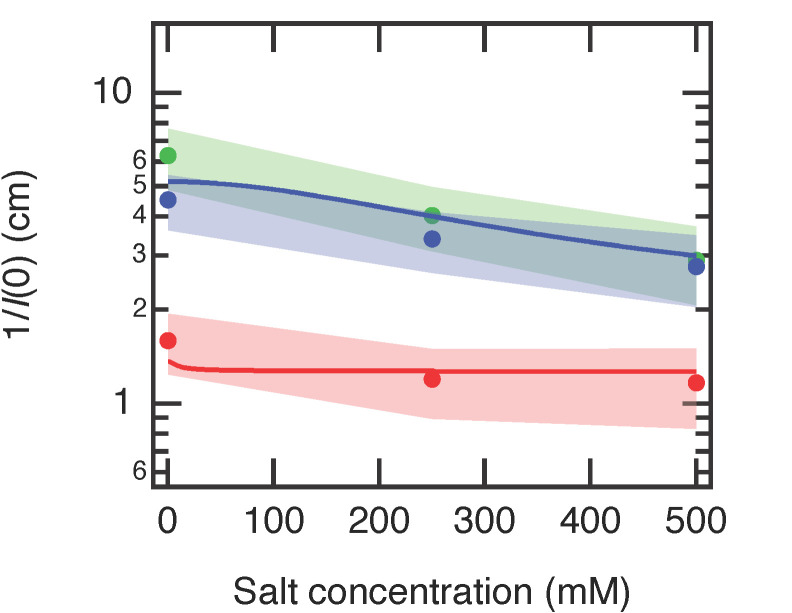
Averaged 1/*I*(0) of PAA gels and solutions as a function of salt (NaCl) concentrations. N0, N50, and N100 denote gels with different neutralization degrees. *I*(0) were determined from the intensity values at *q* = 0.025 Å^−1^ from Figure 4. The solid curves indicate the model curves of K_os_ calculated as a sum of Equations (6) and (7). The red curve is calculated for *α* = 0.005 and the blue curve for *α* = 0.2. The model curve was vertically scaled by a factor of 8 to match 1/*I*(0) values. See the main text for the details of the calculation.

Figure 6 shows the estimated *I*(0) values, plotted as 1/*I*(0) with respect to salt concentration. The *I*(0) values were roughly estimated from the intensity of each profile at *q* = 0.025 Å^−1^ and then averaged for all gels and solutions in each condition. 1/*I*(0) (~*K*_os_) increased when the gels become charged (N0 → N50, N100) and slightly decreased when the salt was added to the gels (S0 → S250, S500). Similar results have been reported in other studies [39,40]. As all gels here were in the same volume, namely the same polymer concentration, the osmotic pressure of mixing, which mainly depends on the polymer concentration, was the same in all gels. Therefore, the other term in the osmotic pressure, Π_ion_, must be responsible for the changes in 1/*I*(0), or *K*_os_. Π_ion_ is present because of the uneven distribution of the mobile ions inside and outside the PAA gels caused by the Donnan equilibrium. According to Equation (3), Π_ion_ increases as the gels become more charged and decreases when adding salts. *K*_os_ has the same behavior as Π_ion_. 

We calculated *K*_os_ = *K*_mix_ + *K*_ion_ using Equations (6) and (7) (Figure 6). The parameters are χ = 0.46, *ϕ* = 0.125, *v*_1_ = 0.018 L mol^−1^, *v*_1_ = 0.0511 L mol^−1^, *z*_p_ = 1, *α* = 0.005 for N0 gel, and 0.2 for N50 and N100 gels. The *K*_os_ curves were scaled by a factor of 8 to match with 1/*I*(0). The model curves qualitatively agree with the experimental results.

**Figure 7:**
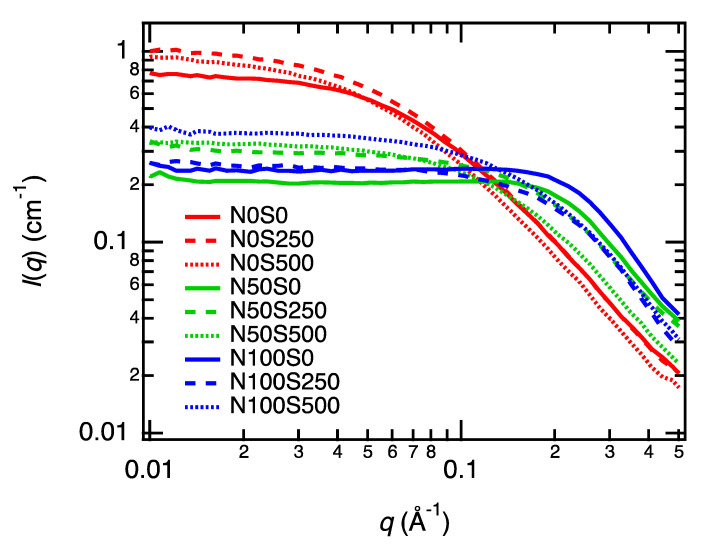
SAXS profiles of the equilibrated equivolume PAA gels, replotted from each panel in Figure 3.

We have replotted the SAXS profiles of the gels at all conditions on one graph (Figure 7). The SAXS profiles changed primarily by tuning the neutralization degree and salt concentrations. The intensity at small *q* values (*q* < 0.02 Å^−1^) decreased, as we discussed above. In addition, a correlation peak appeared at *q* = 0.2 Å^−1^, which is a characteristic scattering profile of the polyelectrolytes [39]. The correlation peak of polyelectrolytes originates from the Coulomb repulsion of line charge on the polymer chain [41]. Although in most previous studies of polyelectrolytes, the correlation peaks were more distinct than what we observed, the vague correlation peak indeed originates from the Coulomb repulsion of the polyelectrolytes. The vagueness of the correlation peak is because the peak appeared at the *q* values of the fractally decaying region of the gels, *q* > 0.1 A^−1^. The correlation peak position depends on the charge density and polymer concentration of polyelectrolytes [42]. From the scaling law of the peak position *q* * with respect to the polymer concentration [43], the correlation peak is supposed to appear at *q* ≈ 0.25 Å^−1^ at the polymer concentration studied here, which is very close to the observed peak position. A similar vague correlation peak was reported at high polymer concentrations [23]. 

The correlation peak of the N100S0 gel is slightly larger than that of the N50S0 gel because the N100S0 gel is more charged. However, the difference in the peak intensity is subtle in comparison with the changes in the neutralization degree (50% → 100%), suggesting the occurrence of the counterion condensation, which suppresses the effective charge density. According to Manning’s theory, when the linear charged density parameter Γ is over 1, the counterion condensation occurs, and the number of free counterions becomes a constant [44]. Γ is defined as *l*_b_/*l*. Here, *l_b_* is Bjerrum length, and *l* is the average distance between neighboring charges. In the fully neutralized PAA gels (N100), *l* is equal to the monomer length, estimated to be 2.5 Å [21]. On the other hand, in the partially neutralized PAA gels (N50), *l* is estimated to be 5.0 Å. Using the *l_b_* value for water, 7.1 Å at 298 K, we obtain Γ = 2.8 for N100 gels and 1.4 for N50 gels. Γ in both conditions exceeds the counterion condensation criteria and explains the observed SAXS profiles of the N50 and N100 gels.

Next, we discuss the salt concentration effect on the profiles. The SAXS profiles of non-neutralized gel (or noncharged gels, N0 series) are almost the same regardless of the added salt concentration. This result is the same as those of neutral gels, where the salt does not change the nanostructures of the gels. Indeed, for N0 samples, most of the carboxyl groups on PAA are not ionized; the dissociation ratio is calculated 5.2 × 10^−3^ by assuming that pKa of PAA is the same with a monomer. Thus, we can regard N0 samples as neutral polymers and do not expect the polyelectrolyte peak at this condition, which explains the SAXS profiles.

In contrast, the scattering profiles of the neutralized gels, N50 and N100 series, changed with the salt addition. Before adding salt, N50S0 (solid green curve) and N100S0 (solid blue curve) gels showed the correlation peak at approximately *q* = 0.2 Å^−1^. However, the correlation peak disappeared after the salt was added. The intensities at small *q* values (*q* < 0.1 Å^−1^) increased with the salt added, but the intensities did not recover to the non-neutralized state, suggesting that the gel nanostructure did not recover the initial state, i.e., the N0S0 condition. The carboxylic acid group concentration in 15 wt% PAA gel is 2.45 mol L^−1^. The molar ratios of sodium chloride to the carboxylic acid group in the S250 and S500 gels are only 10% and 20%, respectively, which are not enough to screen all charged carboxylic acid groups. This estimation accounts for the nonfully recovered small-*q* intensities.

### 3.3. Mechanical Properties of the Post-Ion-Tuned Equivolume PAA Gels 

**Figure 8:**
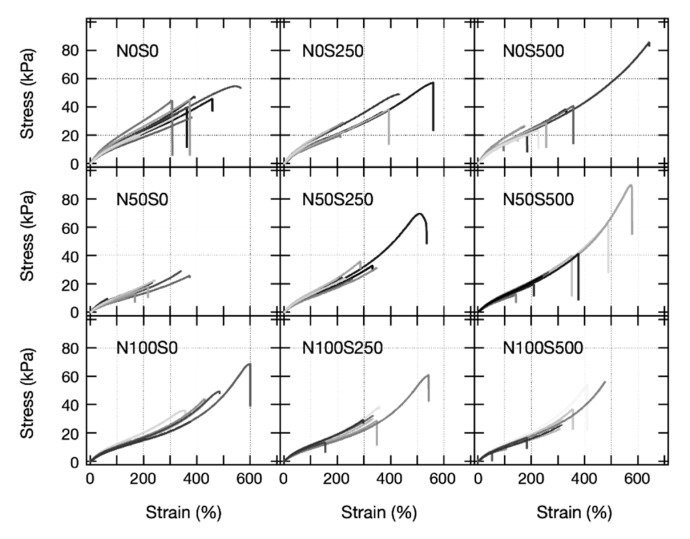
Stress–strain curves of the equivolume PAA gels at various neutralization and salt conditions. Each panel contains at least nine samples’ results.

After revealing the nanostructures of the equivolume PAA gels, we performed tensile tests for the equivolume PAA gels at each neutralization and salt condition. Figure 8 shows stress–strain curves (SS curve) of equivolume PAA gels at each condition. At least nine gel samples were tested for each condition. The variances of the SS curves in each panel are significant because the gels were synthesized by radical copolymerization, which inevitably implements many uncertainties into the gel networks.

**Figure 9:**
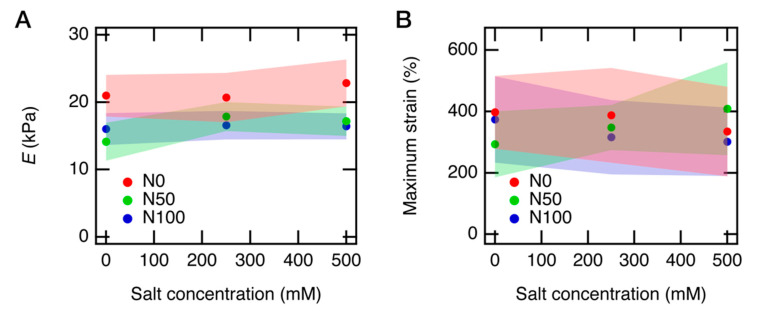
Young’s modulus (*E*) of PAA gels at various neutralization and salt conditions. *E* is plotted as a function of (**A**) salt concentrations and (**B**) neutralization degrees. The average value and the standard deviation for the Young’s modulus *E* and the maximum strain are listed in Table 1 and Table 2.

We estimated the Young’s modulus *E* from the initial slope of each SS curve in all panels in Figure. 8. Its mean value and the standard deviation are shown in Figure 9A as a function of salt concentration. *E* decreased when the gels became charged (N0 → N50, N100) and slightly increased when the salts were added into the gels (S0 → S250, S500). Similar behavior has been reported before for polyelectrolyte gels [17], where the elastic property of gel depends strongly on the charge density of the polyelectrolytes consisting of the gels. The origin of the elasticity of the gels is the configuration entropy of the polymer chains [29]. The decrease of *E* for the charged gels is likely due to the change in the Kuhn segment length, which increases when the polyelectrolytes become charged [45]. Consequently, the number of Kuhn segments per partial chain decreases, resulting in a reduction in the number of chain configurations and the elasticity originated from the entropy. The slight increase in *E* when the salt was added is because the electric potential on the charged chains is screened, and the Kuhn segment lengths decreased. The salt effect may be observed more clearly at a higher salt concentration, which we will perform in a future study.

Rubinstein et al. proposed a theory for the elasticity of polyelectrolyte gels by considering both preparation conditions and measurement conditions of polyelectrolyte gels [46]. Their theory reproduced the result of near equivolume PAA gels by Skouri et al. In this theory, the shear modulus *G* in a weakly stretched regime is described as

(8) $$G=\frac{k_{B}T}{N}c_{m}^{5/6}c_{m}^{0}{}^{1/6}{\left[\frac{1+Buc_{s}/c_{m}}{1+B_{0}uc_{s}^{0}/c_{m}^{0}}\right]}^{1/4}{\left(\frac{B}{B_{0}}\right)}^{1/2}$$

where *k*_B_, *T*, and *N* are the Boltzmann constant, the absolute temperature, and the number of monomers per network strand in the polyelectrolyte gel, respectively. *c*_m_ and *c*_s_ are the molar concentrations of monomers and salt at the measurement condition, respectively. The superscript 0 indicates the preparation state. *u* is ratio of the Bjerrum length to a monomer size, *u* = *l*_B_/*b* = 7.2/2.52 = 2.82. *B* is defined as *Nb*/*L* and written as

(9) $$B={\left(\frac{A^{2}}{u}\right)}^{x}={\left(\alpha^{2}u\right)}^{-x}$$

where *x* is 2/7 in a good solvent. *A* is the number of monomers between uncondensed charges. The reciprocal of *A* is the effective charge fraction after considering the counterion condensation, i.e., *α*. Note, in the original theory, the counterion condensation was not mentioned. In this study, we assumed the initial dissociation ratio of carboxyl groups on PAA, 5.2 × 10^−3^. Since the counterion condensation does not occur at this low dissociation ratio, the effective charges fraction is equal to the dissociation ratio, *α* = 5.2 × 10^−3^, yielding *B*_0_ = 15.0 in the good solvent condition. In our case, *c*_m_ = *c*_m_^0^ and *c*_s_^0^ = 0. The shear modulus at the preparation state, *G*_0_, is given by *G*_0_ = *k*_B_*Tc*_m_/*N*. Combining all discussions, Equation (8) can be rewritten as

(10) $$G=G_{0}{\left(1+Buc_{s}/c_{m}\right)}^{1/4}{\left(\frac{B}{B_{0}}\right)}^{1/2}$$

For noncharged PAA gels, N0 series, *B* is equal to *B*_0_. For charged PAA gels, N50 and N100 series, we need to know *α* at these conditions to calculate *B*. Truzzolillo et al. reported that the free counterion fraction *f* is almost constant when the polymer concentration is higher than the chain overlapping concentration, *c* > *c* * [47], where *f* was approximately 0.148 for a sodium polyacrylate solution (N100S0 condition). Due to the electroneutrality in the solution, *f* times the neutralization degree is equal to *α*; thus, we assume that *α* of N100 samples is 0.148. As *α* is a constant when the counterion condensation occurs, *α* of N50 samples should be 0.148 as well.

The dependence of the effective charge fraction on the salt concentrations in the semidilute regime is unknown. However, a single-chain Monte Carlo simulation showed that the effective charge fraction barely depends on the salt concentration in the case of monovalent salt [48]. Therefore, we assume the effective charge fraction is the same even for S250 and S500 samples. Then, we can calculate the theoretical *E*/*E*_0_ with Equation (10) (Table 3). We assumed that the Poisson’s ratio of PAA gels is 0.5, resulting in a relation between Young’s modulus and shear modulus, *E* = 3*G*. *E*_0_ was 2.1 kPa, and *c*_m_ was 2.45 mol L^−1^.

The theory roughly explains the experimental results, except the salt dependence of N0 gels. We would refrain from a detailed comparison of the theoretical values and the experimental results because we noticed that there are relatively large standard deviations in the measured *E* values, as shown in Figure 9A. 

We also plotted the maximum elongation ratio as a function of salt concentration (Figure 9B). However, because of the substantial standard deviations of the maximum elongation ratios, we could not find a reasonable trend from this result. The significant standard deviation in the maximum elongation ratio, as well as *E* values, is likely because the gel samples were highly slippery, and we needed to firmly compress the gel samples, which might add a bad effect in the elongation tests. The high slipperiness of the gel samples is attributed to the sample preparation of the equivolume PAA gels. As we immersed the gel samples in the highly viscous PAA solutions for the post-ion-tuning, the viscous PAA solutions stickily coated the surface of the gel samples, and we could not perfectly remove them. Although the PAA solution layer on the gel samples is very thin, it still makes the sample more slippery than usual. This problem may be solved if we could decrease the viscosity of the PAA solutions by lowering the molecular weight of linear PAA or putting the gel samples in a dialysis bag to prevent the gel samples from directly contacting the PAA solutions. 

## 4. Conclusions

In this study, by immersing a piece of PAA gel sample in an ion reservoir that contains linear PAA, NaOH, and NaCl at prescribed concentrations, i.e., the post-ion-tuning technique, we, for the first time, prepared equivolume PAA gels in various neutralization and salt conditions. Using this system, we performed SAXS measurements to evaluate the nanostructures of PAA gels and solutions and also performed tensile tests to evaluate the mechanical properties of the gels at each neutralization and salt condition. In the SAXS measurements, the neutralized samples showed characteristic electrostatic scattering profiles, such as the correlation peak and the suppression of the scattering intensity at *q* = 0. These electrostatic features changed with the addition of salt. On the other hand, the Young’s modulus measured with the tensile tests decreased with neutralization degrees increasing. However, the effect of added salt was not clearly observed. Due to the complexity of the polyelectrolyte gels, the further development of the post-ion-tuning technique and a more detailed and systematic study for the resulting equivolume gels may reveal the actual behavior of the gels hidden behind the gels’ complexity. 

## 5. Materials and Methods

### 5.1. Sample Preparation

Polyacrylic acid (PAA) gels were prepared by radical polymerization of 1.5 g of acrylic acid and 12.8 mg of N,N′-methylenebisacrylamide in 8.5 mL of pure water. The final monomer concentration was 15 wt% (2.45 M), and the crosslinker concentration was 0.4 mol% to the monomers. The pregel solution was bubbled with argon gas for 20 min. Then, 3.8 mg of ammonium persulfate and 9 mg of N,N,N′,N′-tetramethylethylenediamine were added into the pregel solution to initiate the polymerization. Then, the pregel solution was kept at 60 °C for more than 2 h to proceed with the polymerization reaction. All reagents were purchased from FUJIFILM Wako Pure Chemical Corporation (Tokyo, Japan) and used without further purification. In the as-prepared PAA gels, all monomers were present as acids, i.e., zero neutralization degree (N0), and no additional salts, NaCl, were added (S0). Therefore, the as-prepared gels were in the N0S0 condition. Here, the capital N denotes the neutralization degree, and the capital S denotes the salt concentration.

### 5.2. Swelling of Prepared Gel

The prepared PAA gel samples were immersed in the linear PAA solutions with the same polymer concentration as the as-prepared gels (15 wt%) but with different salt (NaCl) concentrations (0, 250, and 500 mM; hereafter, we call these S0, S250, and S500, respectively) and neutralization degrees (0, 50, and 100% of the total monomer concentration; N0, N50, and N100). We added NaOH (0, 1.23, and 2.45 M) into the PAA solutions (monomer concentration 2.45 M) to change their neutralization degrees. We used linear PAA with a considerable molecular weight (*M*_n_ = 250 kg mol^−1^) to prevent the linear PAA chains from diffusing into the PAA gels [30]. The volume of the linear PAA solutions used here was at least 20 times larger than that of the gel samples to ensure that the neutralization degree and ion concentrations in the PAA solutions would not change after the post-ion-tuning. The gel samples were immersed in the PAA solutions at least for 24 h.

### 5.3. Observation of Swelling with a Microscope

Disk shape PAA gels with a diameter of 3 mm and thickness of 1 mm were immersed in the PAA solutions. We measured the diameter of the gels with a microscope (BX51-P; Olympus, Japan) equipped with 2x (N.A. 0.06) objective lenses (Nikon, Japan). The camera (WRAYCAM-NF300; Wraymer, Japan) was used to capture images.

### 5.4. Small-Angle X-ray Scattering (SAXS)

SAXS experiment was carried out at BL-6A and BL-10C beamlines of the Photon Factory, High Energy Accelerator Research Organization, KEK (Tsukuba, Japan). The samples were sealed in custom-made cells with silicone rubber sheets and 30 μm glass windows. The sample thickness was 1 mm. The irradiated X-ray wavelength was 1.0 and 1.5 Å. The beam diameter at the sample was approximately 1 mm. The sample-to-detector distance was 1.5 m, and the scattered X-ray was collected by a PILATUS3 2M (DECTRIS Ltd., Baden-Daettwil, Switzerland). The exposure time was 30 s for each sample. All measurements were conducted at ambient conditions. The 1D scattered profile was obtained by circular averaging of the 2D scattered images using a custom-made data reduction package Red2D (https://github.com/hurxl/Red2D, last accessed 24 February 2021) with a scientific data analysis software package (Igor Pro 8, WaveMetrics). Then, 1D SAXS profiles were corrected for exposure time, sample absorption, incident beam flux, and sample thickness. The cell and solvent scattering were subtracted. The calibrations for the absolute intensity and the magnitude of the scattering vector *q* were conducted using a glassy carbon (National Institute of Standards and Technology, Gaithersburg, USA) and silver behenate (Nagara Science, Gihu, Japan).

### 5.5. Tensile Tests

The tensile tests were carried out with a tensile apparatus (EZ-L; SHIMADZU, Kyoto, Japan) equipped with a 50 N load cell. Cylindrical gels with 4 mm in diameter and 35 mm in length were used. The crosshead speed was 60 mm min^−1,^ and all measurements were performed at ambient conditions.

## Figures and Tables

**Table 1:** The average value and the standard deviation for the Young’s modulus *E* of PAA gels at each condition.

	S0	S250	S500
**N0**	20,970 ± 3080	20,690 ± 3660	22,860 ± 3480
**N50**	14,140 ± 2810	17,870 ± 2150	17,180 ± 2810
**N100**	16,020 ± 2370	16,590 ± 2110	16,410 ± 1930

**Table 2:** The average value and the standard deviation for the maximum strain of PAA gels at each condition.

	S0	S250	S500
**N0**	4.0 ± 1.2	3.9 ± 1.5	3.3 ± 1.5
**N50**	2.9 ± 1.1	3.5 ± 0.7	4.1 ± 1.5
**N100**	3.7 ± 1.4	3.2 ± 1.2	3.0 ± 1.1

**Table 3:** Theoretical and experimental *E*/*E*_0_ value for various neutralization degrees and salt concentrations.

Theory				Experimental			
	S0	S250	S500		S0	S250	S500
**N0**	1	1.52	1.76	N0	1	0.986	1.09
**N50**	0.384	0.434	0.472	N50	0.674	0.852	0.819
**N100**	0.384	0.434	0.472	N100	0.764	0.791	0.783

## Data Availability

The data that support the findings of this study are available from the corresponding author upon reasonable request.

## Appendix A

Here, we assume that a polyelectrolyte gel is immersed in a polyelectrolyte solution (reservoir). The osmotic pressure due to the ion distribution at the Donnan equilibrium is given by

(A1) $$\Pi_{\mathrm{ion}}=RT\;\underset{i}{\sum }\left(c_{i}-c_{i}^{’}\right)$$

where *c_i_* and *c_i_*’ are the molar concentrations of the mobile ion of species *i* in the gel and in the reservoir, respectively. *c_i_* and *c_i_*’ are estimated using the equality of the chemical potential at the equilibrium and the electroneutrality in the gel and reservoir.

1. Chemical potential equality

As chemical potential equality of water and each ion species between the gel and reservoir, the osmotic pressure can be given by

(A2) $$\Pi \bar{V_{H_{2}O}}=-RT\ln\frac{c_{H_{2}O}}{c_{H_{2}O}^{’}},\;\;\Pi \bar{V_{{\mathrm{Na}}^{+}}}=-RT\ln\frac{c_{{\mathrm{Na}}^{+}}}{c_{{\mathrm{Na}}^{+}}^{’}},\;\Pi \bar{V_{{\mathrm{Cl}}^{-}}}=-RT\ln\frac{c_{{\mathrm{Cl}}^{-}}}{c_{{\mathrm{Cl}}^{-}}^{’}}$$

where $\bar{V_{H_{2}O}}$, $\bar{V_{{\mathrm{Na}}^{+}}}$, and $\bar{V_{{\mathrm{Cl}}^{-}}}$ are the partial molar volumes of each chemical species, and $c_{H_{2}O}$, $c_{{\mathrm{Na}}^{+}}$, $c_{{\mathrm{Cl}}^{-}}$, $c_{H_{2}O}^{’}$, $c_{{\mathrm{Na}}^{+}}^{’}$, and $c_{{\mathrm{Cl}}^{-}}^{’}$ are the molar concentration of each chemical species in the gel and in the reservoir (noted with ′). By combining the three equations above and assuming $c_{H_{2}O}\approx c_{H_{2}O}^{’}$, we have the equation for osmotic balance:

(A3) $$c_{{\mathrm{Na}}^{+}}c_{{\mathrm{Cl}}^{-}}=c_{{\mathrm{Na}}^{+}}^{’}c_{{\mathrm{Cl}}^{-}}^{’}$$

2. Electroneutrality

The electroneutrality in the gel and reservoir, respectively requires

(A4) $$c_{{\mathrm{Na}}^{+}}+\alpha z_{p}\frac{\phi }{v_{1}}=c_{{\mathrm{Cl}}^{-}}\;c_{{\mathrm{Na}}^{+}}^{’}+\alpha^{\prime }z_{p}\frac{\phi^{\prime }}{v_{1}}=c_{{\mathrm{Cl}}^{-}}^{’}$$

where *α* and *α*’ are the effective charge fractions in the gel and reservoir, respectively. *ϕ* and *ϕ*’ are the polyelectrolytes concentration in the gel and reservoir, respectively. *z*_p_ is the number of chargeable units per monomer, and *v*_1_ is the molar volume of the monomer. *ϕ*/*v*_p_ and *ϕ’*/*v*_p_ are the molar concentrations of the monomers.

By combining Equations (A3) and (A4), we have

(A5) $$c_{{\mathrm{Na}}^{+}}=\frac{\sqrt{\alpha^{2}z_{p}{\left(\frac{\phi }{v_{1}}\right)}^{2}+4c_{{\mathrm{Na}}^{+}}^{’}\left(c_{{\mathrm{Na}}^{+}}^{’}+\alpha^{\prime }z_{p}{\left(\frac{\phi ’}{v_{1}}\right)}^{2}\right)}-\alpha z_{p}\frac{\phi }{v_{1}}}{2}$$

By combining Equations (A1), (A4) and (A5), we have

(A6) $$\frac{\Pi_{\mathrm{ion}}}{RT}|=c_{{\mathrm{Na}}^{+}}+c_{{\mathrm{Cl}}^{-}}-c_{{\mathrm{Na}}^{+}}^{’}-c_{{\mathrm{Cl}}^{-}}^{’}=\sqrt{\alpha^{2}z_{p}{\left(\frac{\phi }{v_{1}}\right)}^{2}+4c_{{\mathrm{Na}}^{+}}^{’}\left(c_{{\mathrm{Na}}^{+}}^{’}+\alpha^{\prime }z_{p}{\left(\frac{\phi ’}{v_{1}}\right)}^{2}\right)}-\alpha^{\prime }z_{p}\frac{\phi^{\prime }}{v_{1}}-2c_{{\mathrm{Na}}^{+}}^{’}$$

Note that when the reservoir volume is much larger than the gel’s, $c_{{\mathrm{Na}}^{+}}^{’}=c_{s}$, where *c*_s_ is the total molar concentration of the added salt. Equation (A6) is the accurate equation for Π_ion_ between the polyelectrolyte gel and the reservoir containing the same polyelectrolytes. However, it is hard to understand the effect of α and α’ from this equation.

By assuming that the polyelectrolyte concentrations and the effective charge fractions in the gel and reservoirs are close, we have

(A7) $$\frac{\Pi_{\mathrm{ion}}}{RT}\approx \left(\alpha -\alpha^{\prime }\right)z_{p}\frac{\phi }{v_{1}}$$

Equation (A7) is a rough estimation of Π_ion_, but we can use it qualitatively to understand the volume change behavior in Figure 2. To distinguish with the Π_ion_ defined in Equation (3), we used ΔΠ_ion_ in the main text to indicate the ionic osmotic pressure between the polyelectrolyte gel and polyelectrolyte solution reservoir.
